# Intergenerational Social Mobility and Health in Later Life: Diagonal Reference Models Applied to the Lothian Birth Cohort 1936

**DOI:** 10.1093/geronb/gbac107

**Published:** 2022-08-11

**Authors:** Matthew H Iveson, Simon R Cox, Ian J Deary

**Affiliations:** Centre for Clinical Brain Sciences, The University of Edinburgh, Edinburgh, UK; Lothian Birth Cohorts, The University of Edinburgh, Edinburgh, UK; Lothian Birth Cohorts, The University of Edinburgh, Edinburgh, UK

**Keywords:** Diagonal reference model, Health, SES, Social mobility

## Abstract

**Objective:**

Although commonly used to model associations between intergenerational social mobility and health, linear regression cannot estimate the contributions of origin, destination, and mobility independently. Nonlinear diagonal reference models (DRMs) have become a popular alternative and have been applied to various health outcomes, though few studies examine the impact of social mobility on later-life health.

**Methods:**

This study revisits health outcomes examined in the Lothian Birth Cohort 1936, using DRMs to assess the association between intergenerational social mobility and satisfaction with life, self-rated health, depression, and mortality from age 68–82 years.

**Results:**

After adjusting for sex, age, education, and childhood cognitive ability, there was no evidence of an association between intergenerational social mobility and later-life health; participants experiencing upward or downward mobility had similar odds of poor health outcomes as non-mobile participants. However, those from higher occupational social classes exhibited lower odds of mortality (*p* = .01), with a stronger contribution of adult (own) than of childhood (father’s) social class (weights = 0.75 vs. 0.25). No other outcomes demonstrated significant associations with socioeconomic position.

**Discussion:**

This adds to evidence that social mobility does not influence variation in later-life health once other factors—including socioeconomic origins and destinations—are accounted for.

Upward intergenerational social mobility—a positive change in the material or social resources of an individual relative to their parents—is one of the key targets of many societies across the world (OECD, 2018). These policy aims are driven by consistently reported associations between higher socioeconomic position and better health and well-being, including lower risk of mortality (Nilsson et al., 2005; Pudrovska & Anikputa, 2014), lower risk of frailty in older age (Gale et al., 2016), lower risk of coronary heart disease (Kittleson et al., 2006), and higher self-reported health (Iveson & Deary, 2017). Beyond position effects, the health impact of upward social mobility is less clear. Upward mobility has been associated with improved health, including higher self-rated physical and mental health (Gugushvili & Präg, 2021) and lower risk of function-limiting conditions (Bartley & Plewis, 2007). However, it has also been associated with poorer health, including higher risk of cardiovascular mortality (Kittleson et al., 2006) and hypertension (Glover et al., 2020). Among hypothesized mechanisms, upward mobility may mitigate the negative health consequences of poorer socioeconomic positions earlier-in-life through improved access to healthcare and healthy behaviors (Pudrovska & Anikputa, 2014), but may also result in stressors related to adjusting to a new socioeconomic position (Chen et al., 2022). Indeed, those experiencing upward mobility from low to high positions typically exhibit poorer health than those in stable-high positions (Pudrovska & Anikputa, 2014).

Importantly, the consequences of social mobility seem to extend into later life. For example, relative to those experiencing stable-low socioeconomic positions, upwardly mobile older adults have been shown to exhibit fewer functional limitations (Luo & Waite, 2005), better self-rated health (Otero-Rodriguez et al., 2011), and lower levels of inflammation (Na-Ek & Demakakos, 2017) across later life. This suggests that promoting upward social mobility among those from poorer socioeconomic backgrounds can have lasting benefits for health.

However, concerns have been raised over previous studies and their use of linear regression models to estimate intergenerational mobility effects (van der Waal et al., 2017). Specifically, mobility effects (i.e., upward, downward, non-mobile) are linearly dependent on origin (i.e., parental) and destination (i.e., own) socioeconomic circumstances, and linear regression models cannot simultaneously estimate all three coefficients accurately. As a result, studies using linear regression models cannot disentangle the contribution of intergenerational social mobility on health and well-being from the contributions of childhood and adult socioeconomic circumstances. This can lead to spurious associations between social mobility and health. For example, van der Waal et al. (2017) noted a significant association between upward mobility and lower odds of obesity (relative to non-mobile individuals) that was present using linear regression models but not using models that separate mobility and position effects.

One alternative method that has recently gained popularity in the field of social mobility is the nonlinear diagonal reference model (DRM; Sobel, 1981), which estimates the contributions of origin and destination socioeconomic position among non-mobile individuals separately to the contribution of mobility. Indeed, previous work comparing DRMs and linear models have found DRMs to be a superior method for studying social mobility in most situations (Präg & Richards, 2019; Steiber, 2019; van der Waal et al., 2017). However, many social mobility studies using DRMs focus on early-life and midlife health outcomes (Jonsson et al., 2017; Schuck & Steiber, 2018); little DRM-based evidence exists regarding differences in later-life health.

In the Lothian Birth Cohort 1936 (LBC1936), a single-year birth cohort of older adults (followed up between age 68 and 82), linear models have identified no significant association between social mobility and either later-life satisfaction or self-rated health (Iveson & Deary, 2017) and no significant association between socioeconomic position (parental or own) and depression status (Iveson et al., 2021). Meanwhile, studies have shown a significant association between higher socioeconomic position and lower later-life mortality risk in the LBC1936 (Fawns-Ritchie et al., 2018). Given the issues with linear models, these studies may misrepresent the importance of intergenerational social mobility and socioeconomic position for these outcomes. In the present study, we apply DRMs to estimate the association between intergenerational social mobility and health and well-being in later life in the LBC1936, revisiting health outcomes previously investigated for their association with social mobility and socioeconomic position. This represents the first use of DRMs in this cohort and the first study to examine the association between intergenerational social mobility and later-life mortality in the LBC1936.

## Method

### Sample

The LBC1936 consists of 1,091 community-dwelling older adults mostly living in the Lothian region of Scotland (Deary et al., 2007; Taylor et al., 2018), recruited at a mean age of 69.53 years (*SD* = 0.83) and followed up over five waves between 2004 and 2018 (Taylor et al., 2018). Recruitment strategy and cohort representativeness are detailed in previous work (Deary et al., 2007). Removing individuals with missing father’s (*N* = 131) or own (*N* = 21) social class resulted in an analytic sample of 941 individuals.

The LBC1936 study is conducted in accordance with the Declaration of Helsinki, with ethical approval from the Research Ethics Committee for Scotland (MREC/01/0/56), the Lothian Research Ethics Committee (LREC/2003/2/29), and the Scotland Research Ethics Committee (07/MRE00/58). Participants provided written informed consent at each wave.

### Measures

#### Occupational social class and social mobility

During Wave 1 (2004–2007) participants reported their father’s highest-status occupation and their own (or spouse’s, if higher) highest-status occupation. Father’s occupation was coded into five classes using the 1950 UK classification index (General Register Office, 1956), and own occupation was coded into six classes using the 1980 UK classification index (Office of Population Censuses and Surveys, 1980). Father’s and own classes were then condensed into a harmonized 4-class categorization ordered from lowest to highest position: Unskilled or partly skilled (combining “unskilled” and “partly skilled” due to small cell counts), Skilled (combining “Skilled manual” and “Skilled non-manual”), Intermediate, and Professional.

Binary variables indicated upward (higher own than father’s class; vs. non-mobile or downward trajectories) or downward (lower own than father’s class; versus non-mobile or upward trajectories) social mobility.

#### Outcomes

Satisfaction with life was measured at Wave 2 (2007–2010) using the Satisfaction With Life Scale (Diener et al., 1985). Responses to five statements (e.g., “In most ways my life is ideal”) using a 7-point scale (1 = “strongly-disagree” to 7 = “strongly-agree”) were scored and summed (max = 35), with higher scores indicating greater satisfaction.

A binary health variable indicated poor self-rated health at any wave (“Poor” or “Fair” health vs. other ratings). At each wave, participants were asked “How would you rate your current health,” responding using a 5-item Likert scale (“Poor,” “Fair,” “Good,” “Very good,” and “Excellent”).

A binary depression variable indicated likely depression at any wave (depressed vs. not depressed). As in previous work (Iveson et al., 2021), depression was defined at each wave as either a Hospital Anxiety and Depression Scale—Depression subscale total score of 8 or more (sensitivity = 74%, specificity = 84%; Wu et al., 2021) or the presence of one or more keywords (related to depression and antidepressants) in a self-reported list of prescribed medication (Iveson et al., 2021).

A binary mortality variable indicated whether individuals had survived to Wave 5 or died earlier in follow-up, as determined from routinely-collected death records provided by the National Health Service.

#### Covariates

Sex and age in years were recorded at Wave 1. Participants also reported the number of years spent in full-time education, which was -transformed (*M* = 0, *SD* = 1).

Childhood cognitive ability (age 11 years) was measured using the Moray House Test No. 12 test of intelligence administered in-school as part of the Scottish Mental Survey 1947 (The Scottish Council for Research in Education, 1949). In line with previous work (Iveson et al., 2021), raw scores (ranging from 0 to 76) were age-adjusted to account for small differences in age at test and then were IQ scaled (*M* = 100, *SD* = 15) to aid interpretability.

### Analyses

Mobility effects were estimated using DRMs (Sobel, 1981); these are nonlinear models that simultaneously estimate the effects of social class origin, destination, and mobility by using non-mobile individuals as a reference group for mobile individuals. The later-life health of mobile individuals is estimated as lying between the average of non-mobile individuals from their origin social class and the average of non-mobile individuals from their destination social class. DRMs summarize mobility effects as two standardized weights (summing to 1) indicating whether mobile individuals best resemble those non-mobile individuals from the origin or destination socioeconomic groups. These can be interpreted as the relative influence of origin and destination class on the outcome; for example, an origin weight of 1 and destination weight of 0 indicates that mobile individuals resemble non-mobile individuals from the origin class.

In the analyses, 3 DRMs were estimated for each outcome and model fit compared. The first model included sex, age (in years, Wave 1), *z*-transformed years of full-time education (Wave 1), and IQ-scaled childhood cognitive ability (age 11). The second model added the diagonal reference term between origin (father’s) and destination (own) occupational social class to estimate separate socioeconomic position effects. The third model further added dummy variables for upward and downward mobility that together indicate social mobility effects. *p*-Values were further FDR-corrected for multiple comparisons across models and outcomes.

Analyses were conducted in R (v4.0.5; R Core Team, 2020) using the “gnm” package (v1.1-1; Turner & Firth, 2020).

## Results

Table 1 shows the descriptive statistics for the analytic sample and for the part of the sample removed during selection due to missing social class variables. The analytic sample were significantly younger, spent longer in education, and reported higher satisfaction than removed individuals. Of the analytic sample, 38% were non-mobile (*N* = 355), 50% were upwardly mobile (*N* = 476), and 12% were downwardly mobile (*N* = 110).

**Table 1.** Descriptives for the Analytic Sample (*N* = 941) and the Sample of Individuals Removed due to Missing Occupational Social Class (*N* = 150)

		Analytic sample		Removed sample		Sample comparison
		*N* (%)	Mean (*SD*)	*N* (%)	Mean (*SD*)	*p*
Sex	Male	465 (49)		83 (55)		0.18
	Female	476 (51)		67 (45)		
	Missing	0 (0)		0 (0)		
Age (years) Wave 1	Mean (*SD*)		69.50 (0.84)		69.74 (0.75)	<0.001
	Missing	0 (0)		0 (0)		
Age (years) Wave 2	Mean (*SD*)		72.47 (0.71)		72.67 (0.74)	0.02
	Missing	167 (18)		58 (39)		
Age (years) Wave 3	Mean (*SD*)		76.24 (0.68)		76.29 (0.67)	0.51
	Missing	313 (33)		81 (54)		
Age (years) Wave 4	Mean (*SD*)		79.31 (0.62)		79.48 (0.61)	0.03
	Missing	447 (48)		94 (63)		
Age (years) Wave 5	Mean (*SD*)		82.00 (0.47)		82.03 (0.51)	0.73
	Missing	551 (59)		109 (73)		
Education (years)	Mean (*SD*)		10.77 (1.13)		10.53 (1.14)	0.01
	Missing	0 (0)		0 (0)		
Father’s social class	Unskilled or partly skilled	164 (17)		2 (1)		0.24
	Skilled	526 (56)		8 (5)		
	Intermediate	185 (20)		7 (5)		
	Professional	66 (7)		2 (1)		
	Missing	0 (0)		131 (88)		
Own social class	Unskilled or partly skilled	34 (4)		10 (7)		0.01
	Skilled	371 (39)		63 (42)		
	Intermediate	364 (39)		38 (25)		
	Professional	172 (18)		18 (12)		
	Missing	0 (0)		21 (14)		
Moray House Test score (age-corrected)	Mean (*SD*)		49.03 (2.23)		48.85 (2.40)	0.45
	Missing	51 (5)		12 (8)		
Satisfaction with life total score	Mean (*SD*)		25.75 (5.78)		23.62 (6.83)	0.01
	Missing	173 (18)		64 (43)		
Poor/fair health status	Poor/Fair health	163 (21)		29 (19)		0.02
	Not Poor/Fair health	611 (79)		63 (42)		
	Missing	167 (18)		58 (39)		
Depression status	Depressed	171 (18)		37 (25)		0.06
	Not depressed	770 (82)		113 (75)		
	Missing	0 (0)		0 (0)		
Deceased status Wave 5	Alive	650 (69)		92 (61)		0.06
	Deceased	291 (31)		58 (39)		
	Missing	0 (0)		0 (0)		

*Notes: p* indicates comparison between the analytic sample (*N* = 941) and individuals removed during sample selection (*N* = 150); Kruskal–Wallis tests are used for numeric variables, and chi-square tests are used for categorical variables.

Table 2 shows the results of the 3 DRMs for each outcome with unadjusted *p*-values. Among baseline covariates, being female was significantly associated with lower odds of poor self-rated health and of death during follow-up, though only the latter survived FDR correction for multiple tests (*p*_FDR_ < .05). Younger age and a 1 *SD* advantage in childhood IQ score were significantly associated with reduced odds of depression during follow-up (both *p*_FDR_ < .05). A 1 *SD* advantage in childhood IQ score also predicted slightly increased odds of death during follow-up though this did not survive FDR correction (*p*_FDR_ = .14). These associations were generally consistent across all 3 models. Nonsignificant associations are given in Table 2.

**Table 2.** Parameter Estimates and Model Fit From the Baseline, Diagonal Reference, and Social Mobility Models Predicting Later-Life Health Outcomes

	Satisfaction with life			Poor self-rated health			Depressive episode			Death		
	Baseline	DRM	DRM + mobility	Baseline	DRM	DRM + Mobility	Baseline	DRM	DRM + Mobility	Baseline	DRM*	DRM + Mobility
	HR	HR	HR	OR	OR	OR	OR	OR	OR	OR	OR	OR
Sex (female)	1.01	1.01	1.01	0.68*	0.67*	0.67*	1.19	1.20	1.20	0.61^***^	0.62^**^	0.61^***^
	[0.98, 1.04]	[0.98, 1.04]	[0.98, 1.04]	[0.47, 0.97]	[0.47, 0.96]	[0.47, 0.97]	[0.84, 1.68]	[0.85, 1.70]	[0.85, 1.70]	[0.46, 0.81]	[0.46, 0.83]	[0.45, 0.82]
Age in years at Wave 1	0.98	0.98	0.98	1.01	1.01	1.01	1.39^**^	1.38^**^	1.38^**^	0.93	0.90	0.90
	[0.97, 1.01]	[0.97, 1.01]	[0.97, 1.01]	[0.80, 1.26]	[0.80, 1.26]	[0.80, 1.26]	[1.12, 1.73]	[1.11, 1.72]	[1.11, 1.73]	[0.77, 1.11]	[0.75, 1.09]	[0.76, 1.07]
Years of **e**ducation (*SD*)	1.01	1.00	1.00	0.87	0.89	0.89	1.03	1.09	1.08	0.91	1.05	1.06
	[0.99, 1.02]	[0.99, 1.02]	[0.98, 1.02]	[0.72, 1.05]	[0.73, 1.08]	[0.72, 1.09]	[0.86, 1.23]	[0.87, 1.33]	[0.88, 1.33]	[0.78, 1.05]	[0.88, 1.25]	[0.90, 1.26]
Childhood IQ score (*SD*)	1.00	1.00	1.00	1.20	1.21	1.21	0.77^**^	0.77^**^	0.77^**^	1.19*	1.17*	1.18*
	[0.99, 1.02]	[0.99, 1.02]	[0.99, 1.02]	[0.99, 1.46]	[0.99, 1.47]	[0.99, 1.47]	[0.64, 0.93]	[0.64, 0.92]	[0.64, 0.92]	[1.01, 1.39]	[1.00, 1.38]	[1.01, 1.38]
Upward mobility	—	—	0.98	—	—	1.04	—	—	0.92	—	—	1.25
			[0.95, 1.02]			[0.67, 1.62]			[0.54, 1.54]			[0.86, 1.82]
Downward mobility	—	—	0.97	—	—	1.14	—	—	0.87	—	—	0.84
			[0.92, 1.03]			[0.56, 2.25]			[0.44, 1.85]			[0.50, 1.40]
Non-mobile coefficients	*B*	*B*	*B*	*B*	*B*	*B*	*B*	*B*	*B*	*B*	*B*	*B*
Unskilled and partly skilled	—	0.53	0.54	—	0.62	0.65	—	0.91	0.91	—	1.96	1.74
Skilled	—	0.47	0.47	—	0.65	0.69	—	0.67	0.57	—	1.73	1.70
Intermediate	—	0.49	0.49	—	0.34	0.36	—	0.69	0.63	—	1.41	1.32
Professional	—	0.51	0.52	—	0.84	0.82	—	0.23	0.20	—	0.68	0.63
Mobile weights	Weight	Weight	Weight	Weight	Weight	Weight	Weight	Weight	Weight	Weight	Weight	Weight
Father’s class	—	0.00	0.00	—	1.00	1.00	—	0.44	0.46	—	0.25	0.00
		[0.00, 0.01]	[0.00, 0.01]		[0.99, 1.00]	[0.99, 1.00]		[0.00, 1.00]	[0.00, 1.00]		[0.00, 0.67]	[0.00, 0.01]
Own class	—	1.00	1.00	—	0.00	0.00	—	0.56	0.54	—	0.75	1.00
		[0.99, 1.00]	[0.99, 1.00]		[0.00, 0.01]	[0.00, 0.01]		[0.00, 1.00]	[0.00, 1.00]		[0.33, 1.00]	[0.99, 1.00]
Model fit												
AIC	4,732.60	4,734.10	4,736.50	763.78	767.75	771.59	838.26	844.53	848.28	1095.10	1090.20	1090.20

*Notes:* AIC = Akaike information criterion; *B* = unstandardized linear regression coefficient; HR = hazard ratio; OR = odds ratio. For diagonal reference models, this includes unstandardized regression coefficients for non-mobile participants in each occupational social class and the relative weights of father’s and own occupational social class for mobile participants. *p*-Values are unadjusted for multiple comparisons. Satisfaction with life model 1 versus 2: Deviance = 4.49, df = 3, *p* = 0.21; Satisfaction with life model 2 versus 3: Deviance = 1.55, df = 2, *p* = .46; self-rated health model 1 versus model 2: Deviance = 2.03, df = 3, *p* = .57; Self-rated health model 2 versus 3: Deviance = 0.16, df = 2, *p* = .92; Depression model 1 versus 2: Deviance = 1.73, df = 4, *p* = .79; Depression model 2 versus 3: Deviance = 0.26, df = 2, *p* = .88; Death model 1 versus 2: Deviance = 12.87, df = 4, *p* = .01; Death model 2 versus 3: Deviance = 2.03, df = 1, *p* = .15. Statistical significance is indicated by

*p < .05,

^**^ *p* < .01, and

^***^ *p* < .001.

Adding the diagonal reference term to estimate socioeconomic position associations did not significantly improve model fit when examining satisfaction with life scores (*p* = .21), odds of poor self-rated health (*p* = .57), or odds of depression (*p* = .79) during follow-up. For these outcomes, model fit was also not significantly improved from the DRM model by adding terms for social mobility direction (*p* = .46, *p* = .92, *p* = .88, respectively).

For mortality odds, adding the diagonal reference term to estimate socioeconomic position associations significantly improved model fit relative to the baseline model (*p* < .01, *p*_FDR_ = .10), but adding terms for social mobility direction did not significantly improve fit further (*p* = .15). In the DRM model, class weights suggested that the mortality odds of mobile individuals better resembled those of non-mobile individuals from their own (destination) social class than from their father’s (origin) social class, though with overlapping CIs. This difference was accentuated in the mobility model indicating almost no association with father’s social class.

## Discussion

The present study revisits later-life health outcomes previously investigated in the LBC1936 (Gale et al., 2016; Iveson & Deary, 2017; Iveson et al., 2021), applying DRMs to disentangle socioeconomic contributions, estimating the contribution of intergenerational social mobility independently of contributions from socioeconomic origin or destination (Sobel, 1981; van der Waal et al., 2017). We observe no evidence that intergenerational social mobility affects later-life satisfaction, self-rated health, depression risk, or mortality risk independent of the contribution from socioeconomic position; mobile individuals experienced similar hazards and odds as immobile individuals from the same class. This is consistent with the few studies that have applied DRMs to older samples (Präg & Richards, 2019; Steiber, 2019). In a cross-sectional study of education and health, Steiber (2019) reports significant social mobility effects in young (30–39 years) and middle-aged (40–49 years) adults, but not in older (60+ years) adults. Steiber (2019) suggests that social mobility effects on health dissipate with age, as the stresses related to the mobility experience become more distant (Präg & Richards, 2019) and as mitigating adaptations are developed (Steiber, 2019). Such adaptations may include positive health behaviors, such as physical activity and healthy diet, as well as factors such as health literacy (Fawns-Ritchie et al., 2018). In the present study, health is assessed several years after economic activity and the destination of measured mobility; participants may have changed their health behaviors and accessed other sources of socioeconomic support (e.g., state pension) to reduce the impact of social mobility on health.

Where intergenerational social mobility was not associated with later-life health independently of socioeconomic position, higher socioeconomic position was associated significantly with reduced mortality odds, though only before correcting for multiple comparisons. Consistent with previous linear regression (Iveson & Deary, 2017; Iveson et al., 2021) and DRM (Präg & Richards, 2019) studies, the relative weights indicated a stronger contribution of adulthood (own) social class than childhood (father’s) social class. Socioeconomic gradients in other later-life health outcomes appear to be explained by demographic (e.g., sex, age) and early-life (e.g., childhood cognitive ability) covariates as highlighted in previous work using this cohort (Gale et al., 2016; Iveson & Deary, 2017; Iveson et al., 2021) and also in other cohorts (Luo & Waite, 2005).

One advantage of using DRMs to separate the contributions of social mobility and socioeconomic position is interpretability, with implications for designing better-informed policy. In the present study, the lack of significant social mobility associations that are independent of socioeconomic origin and destination effects suggests that encouraging upward social mobility may not have a lasting impact on health in later life (Na-Ek & Demakakos, 2017; Steiber, 2019). Instead, to support healthy aging, policy should focus on addressing other sources of health inequality, including improving early-life conditions.

### Limitations

The present sample is subject to health selection; less-healthy individuals may have died prior to follow-up, and this may be partly determined by socioeconomic conditions. Additionally, the DRMs used here are limited to two observations of socioeconomic position; some apparently non-mobile individuals may have changed social class between the two observations (early and later life) and so may not resemble other non-mobile individuals. Furthermore, the present study does not account for how long individuals spent in origin and destination social classes or for other factors that may play an important role in the social mobility–health association such as marital status (Nilsson et al., 2005).

## Conclusion

This study demonstrates that intergenerational social mobility does not predict later-life health beyond the contribution of socioeconomic position and of factors such as sex, age, and early-life cognitive ability.
